# Integrative analysis of circulating microRNAs and the placental transcriptome in recurrent pregnancy loss

**DOI:** 10.3389/fphys.2022.893744

**Published:** 2022-08-05

**Authors:** Naixin Xu, Xuanyou Zhou, Weihui Shi, Mujin Ye, Xianling Cao, Songchang Chen, Chenming Xu

**Affiliations:** ^1^ International Peace Maternity and Child Health Hospital, School of Medicine, Shanghai Jiao Tong University, Shanghai, China; ^2^ Shanghai Key Laboratory of Embryo Original Diseases, Shanghai, China; ^3^ Obstetrics and Gynecology Hospital, Institute of Reproduction and Development, Fudan University, Shanghai, China

**Keywords:** circulating microRNA, recurrent pregnancy loss, biomarker, placenta, pathological pregnancy

## Abstract

Recurrent pregnancy loss (RPL) is a major type of pathological pregnancy that still lacks reliable early diagnosis and effective treatment. The placenta is critical to fetal development and pregnancy success because it participates in critical processes such as early embryo implantation, vascular remodeling, and immunological tolerance. RPL is associated with abnormalities in the biological behavior of placental villous trophoblasts, resulting in aberrant placental function. MicroRNAs (miRNAs) are increasingly being recognized as essential regulators of placental development, as well as potential biomarkers. In this study, plasma miRNAs and placental messenger RNAs (mRNAs) from RPL patients and normal pregnant (NP) controls were sequenced and analyzed. Compared to those in NP controls, 108 circulating miRNAs and 1199 placental mRNAs were differentially expressed in RPL samples. A total of 140 overlapping genes (overlapping between plasma miRNA target genes and actual placental disorder genes) were identified, and functional enrichment analysis showed that these genes were mainly related to cell proliferation, angiogenesis, and cell migration. The regulatory network among miRNAs, overlapping genes, and downstream biological processes was analyzed by protein–protein interactions and Cytoscape. Moreover, enriched mRNAs, which were predictive targets of the differentially expressed plasma miRNAs miR-766-5p, miR-1285-3p, and miR-520a-3p, were accordingly altered in the placenta. These results suggest that circulating miRNAs may be involved in the pathogenesis of RPL and are potential noninvasive biomarkers for RPL.

## Introduction

Recurrent pregnancy loss (RPL) is one of the most complex and challenging scenarios in reproductive medicine and is defined as the failure of two or more pregnancies (Bolondi et al., 2016). The prevalence of RPL is difficult to estimate; however, most studies claim that 1–4% of women are affected (Rai and Regan, 2006; Rasmark Roepke et al., 2017; Magnus et al., 2019). RPL can be attributed to heredity, structure, infection, endocrine issues, immunity, or unknown causes (Rai and Regan, 2006). The placenta is the first organ to form during pregnancy and is critical for embryonic development and successful pregnancy because it participates in critical processes such as early embryo implantation, vascular remodeling, and immunological tolerance. Although the specific pathogenesis of RPL is unclear, it is commonly recognized that the placenta plays an important role in this process. The origin and development of the placenta are closely related to the proliferation, differentiation, and functional state of placental trophoblasts (Lala et al., 2021). Pathological pregnancies, such as preeclampsia (PE) and pregnancy loss, are thought to be mediated by placental trophoblast dysfunction (Huppertz, 2019; Lokeswara et al., 2021). Thus, it is crucial to understand the molecular mechanisms underlying trophoblast turnover and identify corresponding biological targets.

MiRNAs are endogenous, short noncoding single-stranded RNAs with an average length of 22 nucleotides. MiRNAs regulate gene expressions in a sequence-specific manner mainly through posttranscriptional inhibition or degradation of messenger RNAs. Approximately 600 miRNAs are expressed in the human placenta and play key roles in placental development and function by regulating the expressions of genes involved in trophoblast proliferation, differentiation, invasion, migration, apoptosis, and angiogenesis (Mouillet et al., 2015; Chen et al., 2021). A number of differentially expressed miRNAs have been confirmed in the placenta in pathological pregnancies, including RPL, PE, intrauterine growth restriction, and preterm delivery (Morales-Prieto et al., 2014; Tian et al., 2020; Tang et al., 2021). Besides their effects on intracellular silencing, placental-derived miRNAs are appealing because they can be released into maternal circulation by being packaged within extracellular vesicles (Luo et al., 2009; Morales-Prieto et al., 2020). It is possible to provide a hormone-like way for different maternal cells to communicate with each other and even between the mother and the fetus (Xu et al., 2021). Therefore, the differential expressions of miRNAs in maternal circulation can indicate potential placental pathology and can be used as biomarkers for the early detection of pregnancy complications (Zhao et al., 2013; Tian et al., 2020; Munjas et al., 2021).

Although several investigations have been performed on the expressions and functions of miRNAs in the placentas of patients with RPL, few researchers have conducted joint studies on the regulatory network of plasma miRNAs and placental mRNAs. In this study, we combined plasma miRNA sequencing with placental mRNA sequencing for the first time. The target genes of differentially expressed plasma miRNAs and placental mRNAs were cross-screened for further gene function analysis and protein–protein interaction (PPI) regulatory network construction. We focused on 10 hub genes and 7 central miRNAs. Functional analysis and literature reviews showed that they were related to pathological pregnancy by participating in cell migration, apoptosis, and angiogenesis. We aimed to elucidate the miRNA–mRNA regulatory network in RPL and provide a promising target for the diagnosis and treatment of RPL.

## Materials and methods

### Study population

Patients with RPL were recruited for this study at Shanghai Jiao Tong University School of Medicine International Peace Maternity and Child Health Hospital from December 2018 to December 2019. All participants were thoroughly examined to rule out any known causes of RPL. Patients with the following characteristics were excluded from the study: 1) genital malformation on pelvic examination and ultrasound, 2) abnormal immune function, 3) endocrine or metabolic disease symptoms, 4) infectious disease, 5) history of family genetic diseases, 6) pathogenic copy number variant (CNV) sequencing in chorionic villous tissue, and 6) other identified causes of miscarriage.

The normal pregnant (NP) group included normal early pregnant healthy women who underwent surgical abortion according to their wishes. We also excluded patients who could not sustain pregnancy due to their physical condition or patients whose fetuses had been identified with malformations or congenital illnesses.

The study was authorized by the Medical Ethics Committee of the International Peace Maternity and Child Health Hospital, China Welfare Institute, and all participants provided informed consent in accordance with ethical norms.

### Biological samples

Placental chorionic villous tissue approximately 5 mm in size was collected during dilation and curettage. Under the dissecting microscope, chorionic villous samples were isolated from maternal decidua after being repeatedly washed with sterile saline. The clean tissue was immersed in RNAlater (ThermoFisher, AM7024) and immediately stored at −80°C.

Approximately 5 ml of venous blood was collected in an EDTA peripheral blood anticoagulant tube before the surgery. The blood was gently inverted and mixed 6–8 times after collection and centrifuged at 1600 × *g* for 10 min. The separated plasma was centrifuged at 12000 × *g* for 10 min. After two rounds of centrifugation, the plasma (approximately 2 ml) was stored at −80°C.

### RNA isolation from villous samples

An mRNA isolation kit was used to isolate total RNA from placental tissues according to the manufacturer’s instructions (Ambion, Invitrogen, AM1561). The NanoDrop 2000 spectrophotometer was used to assess the purity and quantity of RNA (Thermo Scientific, United States). The Agilent 2100 Bioanalyzer (Agilent Technologies, Santa Clara, CA, United States) was used to evaluate RNA integrity.

### RNA isolation from maternal plasma samples

Total mRNA was extracted from 200 μl of plasma using the QIAGEN miRNeasy Serum/Plasma Advanced Kit (Qiagen, cat. # 217204) according to the manufacturer’s instructions. The NanoDrop 2000 was used for total RNA quantification (Thermo Fisher Scientific Inc., United States). The RNA integrity was determined using an Agilent 2100 Bioanalyzer (Agilent Technology, United States).

### Library preparation and sequencing procedures

In the screening process, we investigated a pooled RNA sample extracted from villous and plasma samples of three RPL patients and three NP controls, matched for age and other clinical characteristics listed in Table 1.

**TABLE 1:** Clinical characteristics of patients recruited.

	RPL	NP	*p*-value
*N*	20	21	-
Age (years)	31 (26,41)	30 (22,40)	NS
Gestational age (weeks)	8 (7,9)	7 (7,8)	NS
Maternal prepregnancy BMI (kg/m^2^)	20.3 (16.0,27.1)	19.4 (15.2,22.5)	NS
Smoking	3 (0.15%)	2 (0.10%)	NS
Cycle length (days)	30 (28,35)	30 (27,34)	NS
Previous pregnancy losses	2 (2,3)	0 (0,0)	-

Data are shown as median [range] or n (%). NS, not significant; RPL, recurrent pregnancy loss; NP, normal pregnancy; BMI, body mass index.

The mRNA libraries of villous tissues were constructed using the TruSeq Stranded mRNA LT Sample Prep Kit (Illumina, San Diego, CA, United States) according to the manufacturer’s instructions. The Illumina HiSeq X Ten platform was used to sequence the libraries, and 150-bp paired-end reads were produced. Each sample produced approximately 49.52 M raw readings. Trimmomatic was used to initially process the raw data (raw readings) in the fastq format, through the removal of low-quality reads to produce clean reads (Bolger et al., 2014). Then, for each sample, 48.57 M clean readings were kept for later analyses. HISAT2 was used to map the clean reads to the human genome (GRCh38) (Kim et al., 2015). The FPKM of each gene was computed using Cufflinks, and the HTSeq-count was used to determine the read counts of each gene (Trapnell et al., 2010; Roberts et al., 2011; Anders et al., 2015). All procedures were performed by OE Biotech Co., Ltd. (Shanghai, China).

Following the manufacturer’s guidelines, 1 ug of total RNA from each plasma sample was utilized for the small RNA library building using TruSeq Small RNA Sample Prep Kits (Cat. No. RS-200–0012, Illumina, United States). In a nutshell, the whole RNA was ligated to adapters at both ends. The adapter-ligated RNA was then reverse-transcribed to cDNA and PCR amplification was conducted. Small RNA libraries were created from PCR products ranging in size from 140 to 160 bp. The quality of the library was evaluated using DNA High Sensitivity Chips on the Agilent Bioanalyzer 2100 instrument. Base-calling was used to transform the raw reads from the basic reads into sequence data. The readings containing 5′ primer contamination and poly(A) were eliminated after low-quality reads were filtered. To acquire clean reads, raw data were filtered to remove reads with the 3′ adapter and insert tag as well as reads that were either 15 nt or 41 nt in length. For the primary analysis, the length distribution of the clean sequences in the reference genome was determined. Small nuclear RNAs, rRNAs, tRNAs, and other terms were used to annotate noncoding RNAs. Following the alignment of these RNAs, a Bowtie search was conducted against Rfam v.10.1 (http://www.sanger.ac.uk/software/Rfam) (Langmead et al., 2009). By matching the known miRNAs to the miRBase v22 database, the known miRNAs were located, and the known miRNA expression patterns in various samples were examined (Griffiths-Jones et al., 2008). Next, miRDeep2 was used to predict new miRNAs from unannotated readings (Friedländer et al., 2012). OE Biotech Co., Ltd., performed the small RNA sequencing and analysis (Shanghai, China).

### Bioinformatic analysis

Differentially expressed mRNAs and miRNAs were used to identify the DESeq (2012) functions with estimateSizeFactors and nbinomTest. The threshold for significantly differential expression was established as a *P* or *q* < 0.05 and |log2-fold change| > 1.

### Enrichment analysis

Target genes of miRNAs were predicted using the datasets collected from miRTarBase, miRWalk, and Diana Tools (Maragkakis et al., 2011; Chou et al., 2018; Sticht et al., 2018). The genes involved in overlapping between miRNA target genes and actual differentially expressed placental genes were referred to as overlapping genes. To identify the potential relevant functional annotation and pathway enrichment analysis of overlapping genes, Gene Ontology (GO) and Kyoto Encyclopedia of Genes and Genomes (KEGG) were used. Analyses were conducted using the Database for Annotation, Visualization, and Integrated Discovery [DAVID3], with a significance level of *p* < 0.05 (Dennis et al., 2003).

### Protein–protein interaction network

The PPI data were obtained from the STRING database (https://string-db.org/) (Szklarczyk et al., 2017). We built the interaction network with the STRING database and selected PPI pairs based on a combination score > 0.4 to assess the PPIs of overlapping genes. Then, the PPI network was analyzed by Cytoscape visualization (Shannon et al., 2003). The Cytoscape program CytoHubba was used to choose the hub genes.

### Building a miRNA–mRNA regulatory network

A regulatory network of miRNA–mRNA interactions was constructed by Cytoscape to show the interaction between differentially expressed circulating miRNAs and overlapping genes.

### Cumulative distribution plots

The fold change between target and nontarget genes of differentially expressed plasma miRNAs was compared using the cumulative distribution function. The Mann–Whitney U test was used to evaluate whether the log2fold change in target genes in the placentas of RPL patients was significantly changed compared with that of nontarget genes.

### RT-qPCR validation analysis

To confirm the reliability of the sequencing results, crucial dysregulated villous mRNAs and plasma miRNAs were subsequently validated in all samples (20 RPL patients and 21 healthy controls). According to the manufacturer’s instructions, mRNA and miRNA were reverse-transcribed using PrimeScript RT reagent and Mir-X miRNA First-Strand Synthesis Kit (Takara, Japan). The TB Green qPCR Master Mix (Takara, Japan) was used to perform quantitative real-time PCR (qPCR) analyses according to the manufacturer’s instructions. The quantities of mRNA and miRNA transcripts were standardized to the housekeeping genes GAPDH and U6, respectively. Relative mRNA and miRNA expressions were plotted as 2−ΔΔct values. The primer sequences used for qPCR are shown in Table 2.

**TABLE 2:** Sequences of the primers used in qRT-PCR experiments.

mRNAs	Primers	miRNAs	Primers (Universal reverse primer provided by kit)
FYN	F:TACTCAAAAGTGGGGCGTTC	hsa-miR-145-5p	F:AACCTCCGTCCAGTTTTCCCA
	R:ACGGGAGGTTCACAATCAAG		
KDR	F:CAGCATCACCAGTAGCCAGA	hsa-miR-493-5p	F:AATCGGCGTTGTACATGGTAGG
	R:ATTTCCCACAGCAAAACACC		
PECAM1	F:TATTTTCCAAGCCCGAACTG	hsa-miR-1285-3p	F:ACGACAATCTGGGCAACAAAGT
	R:TCACCTTCACCCTCAGAACC		
PDGFRB	F:CACTGCCTGTCCCCTATGAT	hsa-miR-766-5p	F:AACACGTGAGGAGGAATTGGTG
	R:TCAGAATCCACCTCCCTGTC		
		U6	Primer provided by kit

### Statistical analysis

For statistical analysis, GraphPad Prism version 8 software (GraphPad Software, San Diego, United States) was used. Differences between groups of continuous variables were tested using a parametric two-tailed t test or Mann–Whitney U test when the normality assumption was not met. The chi-squared test was used for comparison of categorical variables. Statistical significance was accepted at *p* < 0.05.

## Results

### Clinical characteristics of the patients

In the current study, 20 RPL patients and 21 NP controls who fulfilled the inclusion criteria were included. The demographic information of the participants is shown in Table 1. There were no significant differences in maternal age, gestational age, maternal prepregnancy body mass index, smoking status, or cycle length between the two groups.

### Differentially expressed circulating miRNAs and placental mRNAs

**FIGURE 1:**
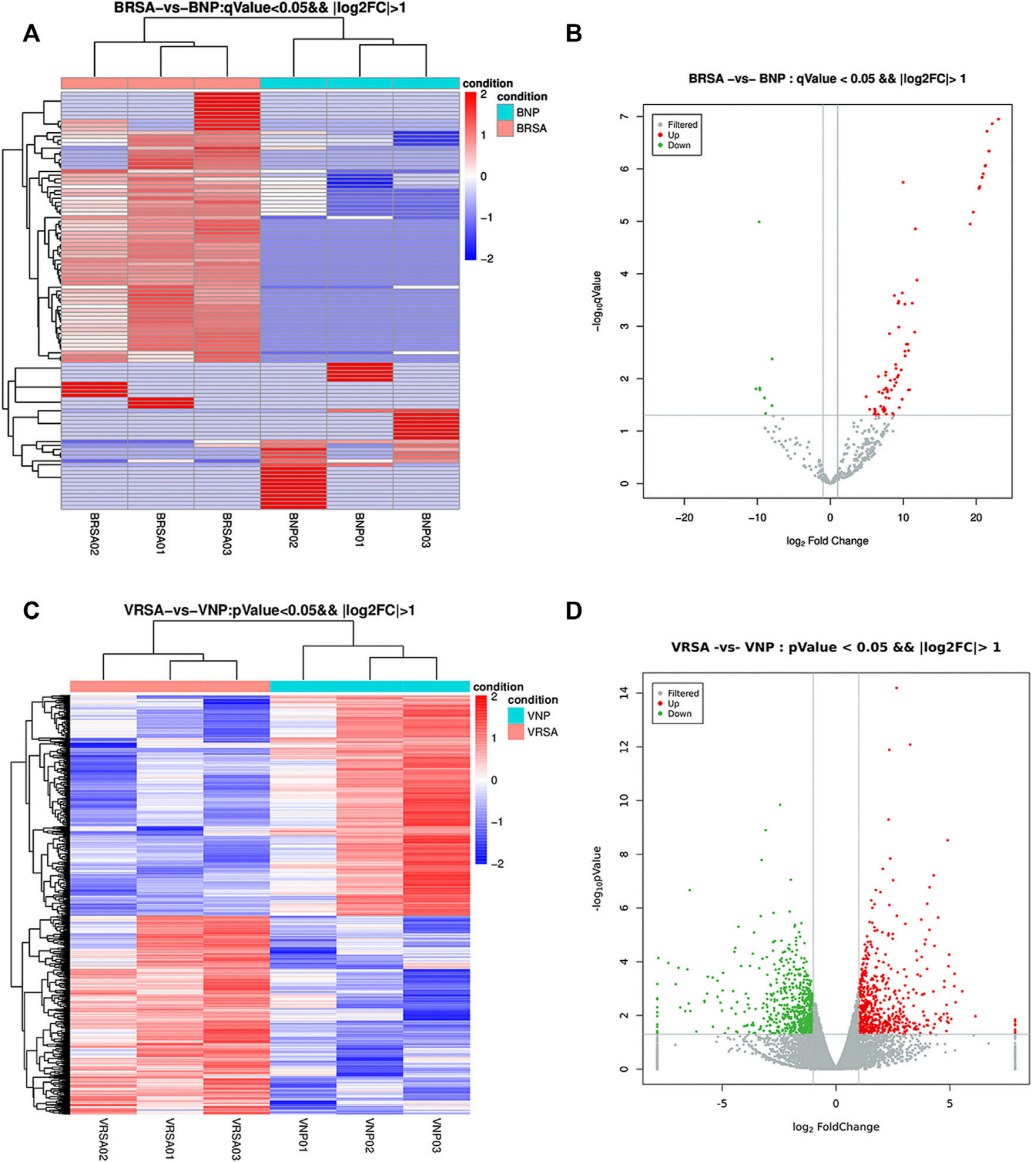
Expression profiles of circulating miRNAs and placental mRNAs in the NP and RPL groups (*n* = 3 per group). **(A)** heatmap of circulating miRNAs. **(B)** volcano plot of circulating miRNAs. **(C)** heatmap of placental mRNAs. **(D)** volcano plot of placental mRNAs.

Considering a |log2-fold change| >1 and a *q* < 0.05, a total of 108 circulating miRNAs showed differences. A total of 77 miRNAs were upregulated and 31 were downregulated in the RPL group compared with the NP group (Figures 1A and B). Placental villous mRNA expression profiles with the same population were investigated. Considering a |log2-fold change| > 1 and *p* < 0.05, a total of 1199 differentially expressed transcripts were identified in placental tissue. Among them, 568 mRNAs were upregulated and 631 were downregulated (Figures 1C and D). The detailed miRNAs and mRNAs sequencing results are in Supplementary Table S1 and Supplementary Table S2.

### Identification of the candidate target genes of differentially expressed miRNAs

**FIGURE 2:**
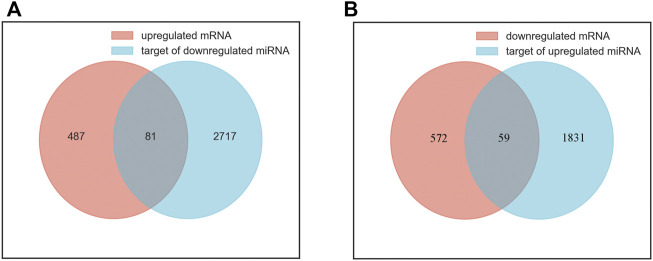
Identification of overlapping genes. **(A)** the intersection of upregulated mRNAs (red) and target genes of downregulated miRNAs (blue). **(B)** the intersection of downregulated mRNAs (red) and target genes of upregulated miRNAs (blue).

A total of 5607 target genes were identified, including 1890 targets of upregulated miRNAs and 2798 targets of downregulated miRNAs. There were overlaps between anticipated target genes and the measured mRNAs, adding to the evidence for the involved genes. We performed a joint analysis of the putative target genes of differentially expressed miRNAs and differentially expressed mRNAs. Eighty-one genes overlapped between the upregulated mRNAs and the target genes of the downregulated miRNAs were called upregulated overlapping genes. Fifty-nine genes overlapped between the downregulated mRNAs and the target genes of the upregulated miRNAs were called downregulated overlapping genes (Figure 2). A total of 140 overlapping genes were identified (Supplementary Table S3).

### Functional annotation and pathway enrichment analysis

**FIGURE 3:**
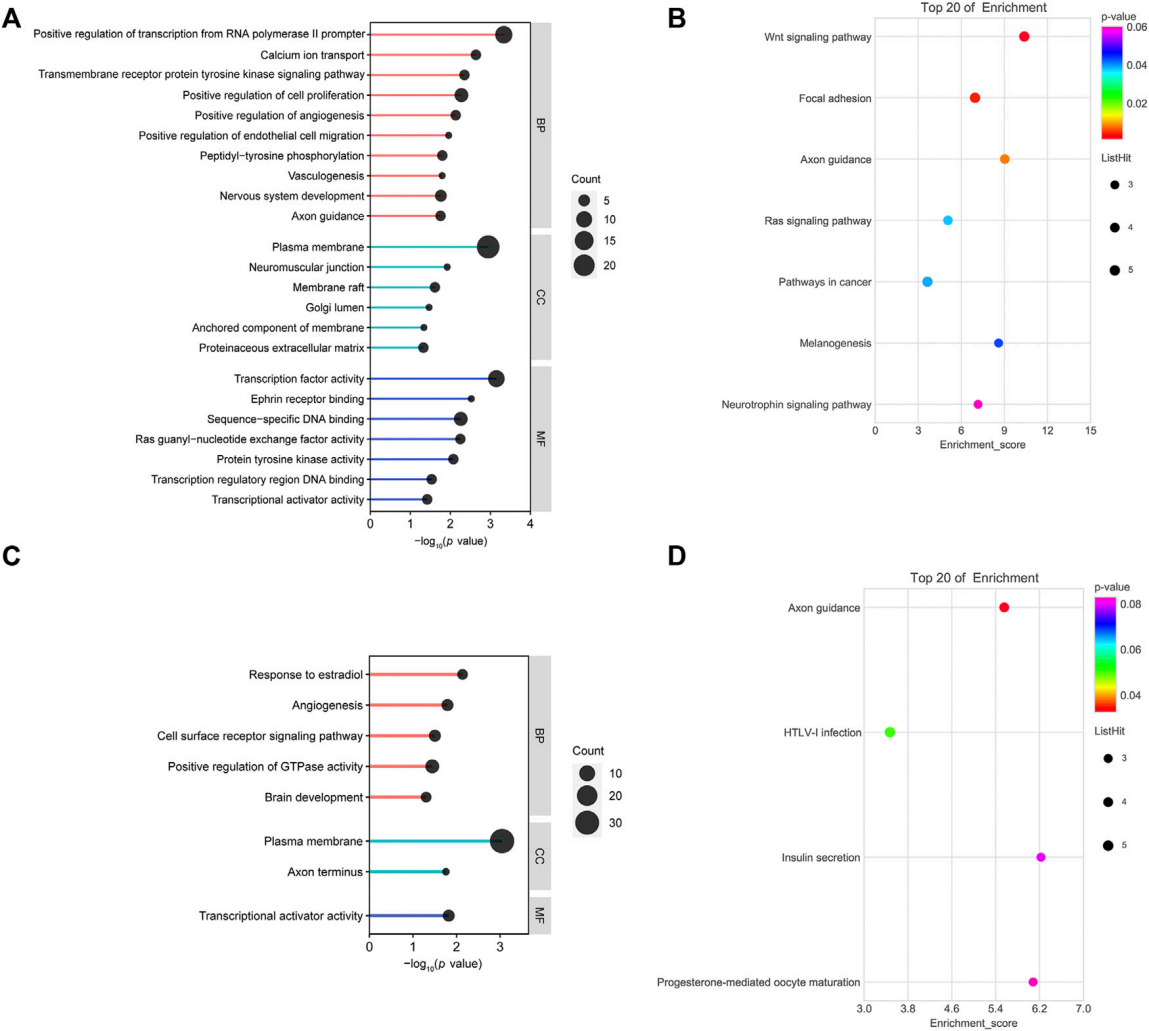
Functional analysis of overlapping genes: **(A)** gene ontology (GO) analysis of downregulated overlapping genes. **(B)** Kyoto Encyclopedia of Genes and Genomes (KEGG) pathway analysis of downregulated overlapping genes. **(C)** GO analysis of upregulated overlapping genes. **(D)** KEGG pathway analysis of upregulated overlapping genes.

GO and KEGG pathway analyses were performed on the up/downregulated overlapping genes. GO consists of three categories: molecular cellular components, biological processes (BPs), and molecular functions (MFs). As shown in Figure 3A, BP-related results showed that downregulated overlapping genes were mainly involved in cell proliferation, angiogenesis, vasculogenesis, and cell migration, which are BPs that are closely related to abortion. In addition, the results of the KEGG pathway analysis showed that the downregulated overlapping genes were mainly enriched in the Wnt signaling pathway, focal adhesion, and axon guidance (Figure 3B). BP-related results showed that upregulated overlapping genes were particularly enriched in responses to estradiol, angiogenesis, and the cell surface receptor signaling pathway (Figure 3C). The KEGG pathway analysis results showed that the upregulated overlapping genes were mainly enriched in axon guidance (Figure 3D).

### Protein–protein interaction network

**FIGURE 4:**
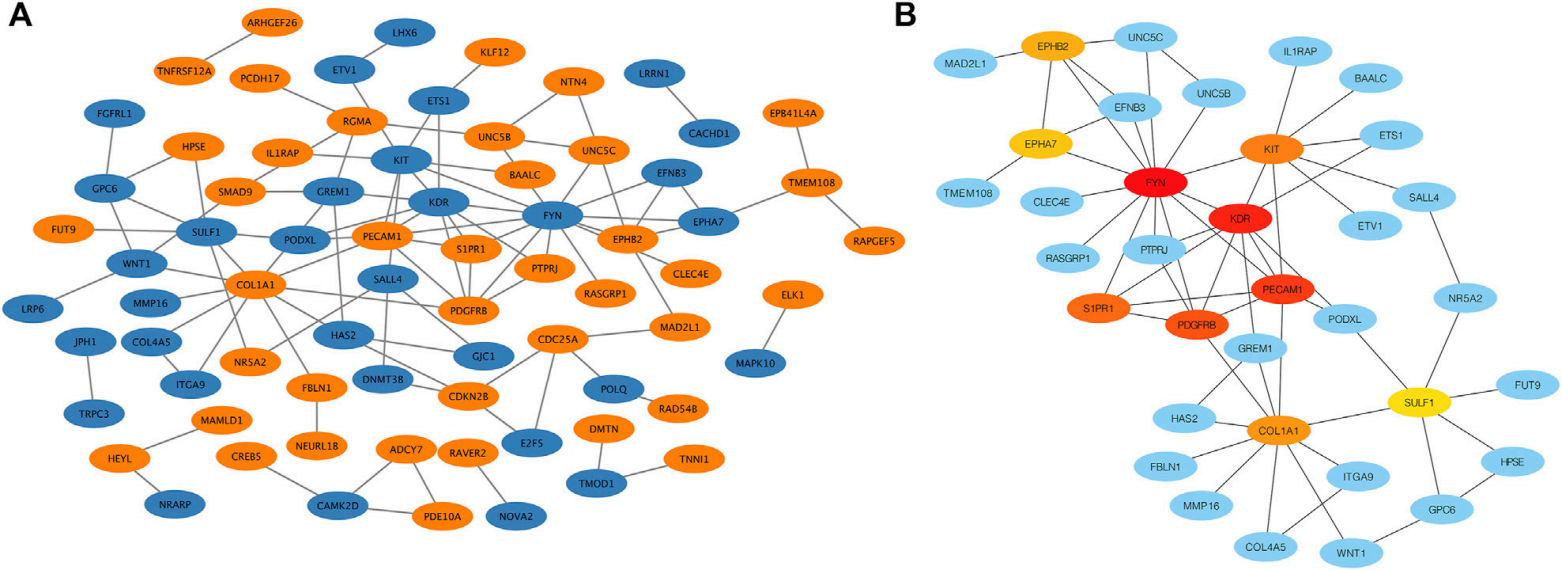
Protein–protein interaction (PPI) network. **(A)** PPI network of overlapping genes. The blue circle represents downregulated overlapping genes, and the orange circle represents upregulated overlapping genes. **(B)** PPI network of top 10 overlapping genes (hub genes). The darker the circle, the greater the connectivity score.

**FIGURE 5:**
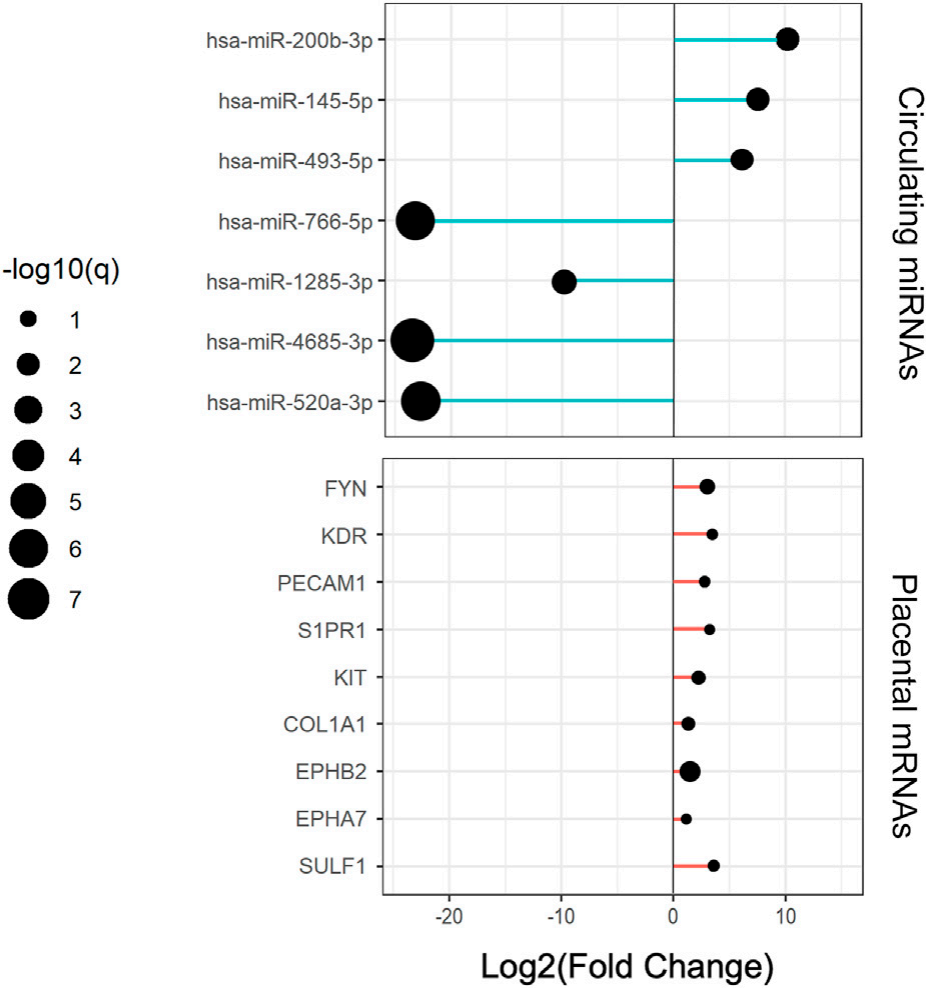
Expression changes of circulating central miRNAs and placental hub genes in RPL relative to the NP group.

To explore the interaction network between target proteins and the core regulatory genes, the STRING method was used to predict the interactions of 140 overlapping genes at the protein level. In the PPI network of the overlapping genes, a total of 134 nodes and 99 edges were mapped (Figure 4A). We further identified the top 10 hub genes assessed by CytoHubba (Figure 4B). The top 10 hub genes were *FYN*, *KDR*, *PECAM1*, *PDGFRB*, *S1PR1*, *KIT*, *COL1A1*, *EPHB2*, *EPHA7*, and *SULF1* (Table 3). The differential expressions of hub genes between the two groups are shown in Figure 5.

**TABLE 3:** Top 10 overlapping genes (hub genes) with highest connectivities.

Rank	Name	Score	Description
1	*FYN*	48	src family tyrosine kinase
2	*KDR*	41	kinase insert domain receptor
3	*PECAM1*	34	platelet and endothelial cell adhesion molecule 1
4	*PDGFRB*	32	platelet-derived growth factor receptor beta
5	*S1PR1*	24	sphingosine-1-phosphate receptor 1
6	*KIT*	12	receptor tyrosine kinase
7	*COL1A1*	10	collagen type I alpha 1 chain
8	*EPHB2*	9	EPH receptor B2
9	*EPHA7*	7	EPH receptor A7
10	*SULF1*	6	sulfatase 1

### miRNA–overlapping gene regulatory network establishment

**FIGURE 6:**
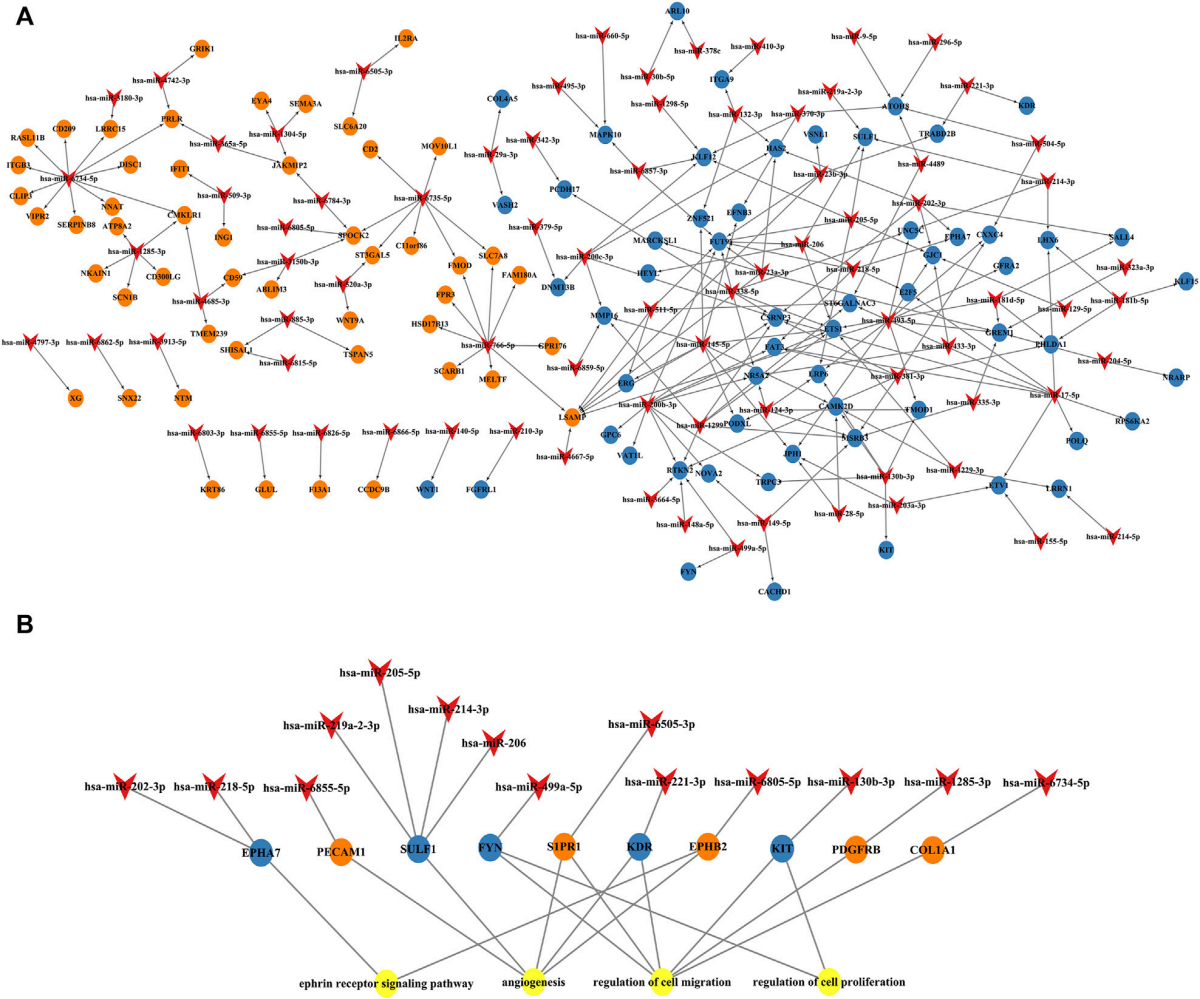
miRNA and mRNA network. **(A)** Construction of miRNA-overlapping genes regulatory network. The diamond represents differentially expressed circulating miRNAs, the blue circle represents downregulated overlapping genes, and the orange circle represents upregulated overlapping genes. **(B)** Predicted biological processes (BPs) of GO involving top 10 hub genes regulated by differentially expressed plasma miRNAs in RPL patients.

Cytoscape was used to visualize the miRNA target gene regulatory network between overlapping genes and miRNAs (Figure 6A). Among the upregulated miRNAs, mir-200b-3p regulates 10 downregulated target genes and was the upregulated miRNA with the highest number of target genes. Both miR-145-5p and miR-493-5p regulate 9 downregulated overlapping genes. MiR-766-5p, miR-1285-3p, miR-4685-3p, and miR-520a-3p regulate 11, 11, 9, and 7 overlapping genes respectively and were the top four downregulated miRNAs with the largest number of target genes. We identified 7 central differentially expressed miRNAs (upregulated mir-200b-3p, miR-145-5p, and miR-493-5p; downregulated miR-766-5p, miR-1285-3p, miR-4685-3p, and miR-520a-3p) (Table 4). The differential expressions of central miRNAs in the plasma between the two groups are shown in Figure 5.

**TABLE 4:** Seven central differentially expressed miRNAs and target overlapping genes.

MiRNAs	Overlapping genes
hsa-miR-200b-3p	*CSRNP3, ETS1, FAT3, FUT9, GPC6, NOVA2, NR5A2, RTKN2, TRPC3, VAT1L*
hsa-miR-145-5p	*CAMK2D, EFNB3, ERG, ETS1, JPH1, LSAMP, MMP16, PODXL, ZNF521*
hsa-miR-493-5p	*CXXC4, GFRA2, GJC1, GREM1, JPH1, LRP6, LSAMP, TMOD1, UNC5C*
hsa-miR-766-5p	*ADGRG5, BAALC, FPR3, MAMLD1, MY O 1A, NR5A2, PSG4, RASGRP1, TMEM108, TNNI1, USP46*
hsa-miR-1285-3p	*AFF3, ARNT2, CREB5, DMTN, EPB41L4A, HEYL, PDGFRB, SH3TC2, SLC25A23, TOX2, TRIM59*
hsa-miR-4685-3p	*CD59, CREB5, KCNA6, MEX3A, PTPRJ, RASL10B, S1PR1, TNFRSF12A, TOX2*
hsa-miR-520a-3p	*ARL4C, CDC25A, CREB5, KLF12, OR51E1, RAPGEF5, RGMA*

Next, we established an interaction network among the differentially expressed miRNAs, hub genes, and the BPs of GO analysis (Figure 6B). Hub genes were extensively regulated by circulating miRNAs and are involved in BPs such as the ephrin receptor signaling pathway, angiogenesis, cell migration, and cell proliferation of placental villi.

### Downregulation of circulating miRNAs leads to global upregulation of placental targets

**FIGURE 7:**
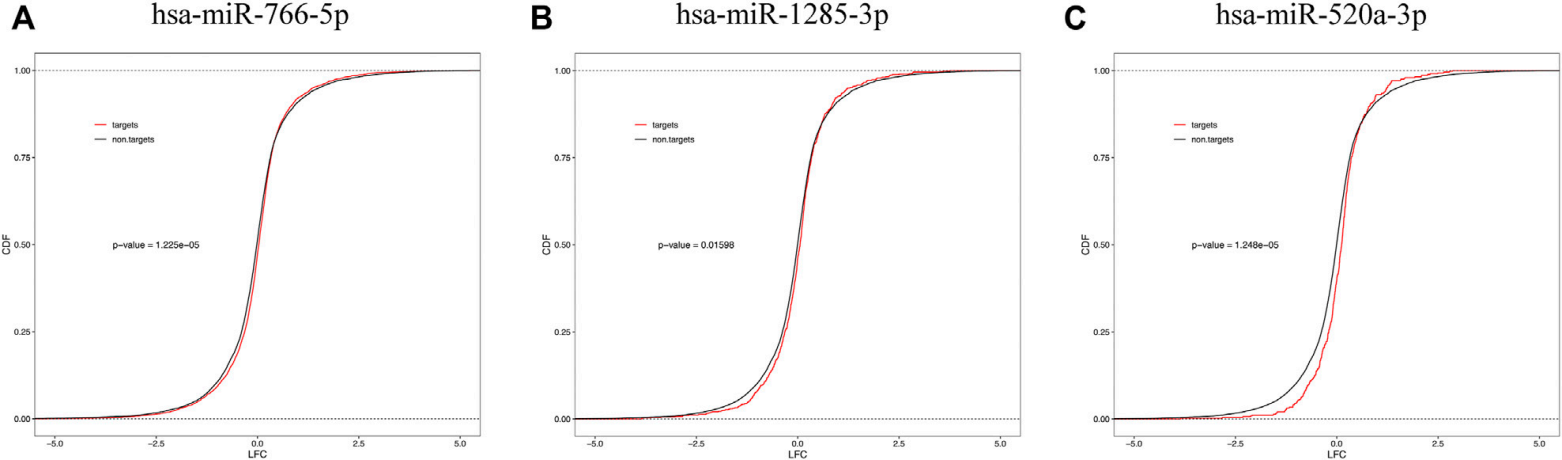
Cumulative frequency of mRNAs log2 fold change based on miRNA target status. mRNA targets of downregulated plasma miRNAs [miR-766-5p **(A)**, miR-1285-3p **(B),** and miR-520a-3p **(C)**] are upregulated in the placental tissue.

We examined the cumulative distribution function of the mRNA targets of central differentially expressed miRNAs. As shown in Figure 7, the predicted targets of downregulated miRNAs (miR-766-5p, miR-1285-3p, and miR-520a-3p) were upregulated in the placenta (a righthand shift in the target mRNA compared with the nontarget mRNA indicates increased mRNA expression).

### Validation of differentially expressed miRNAs and mRNAs

**FIGURE 8:**
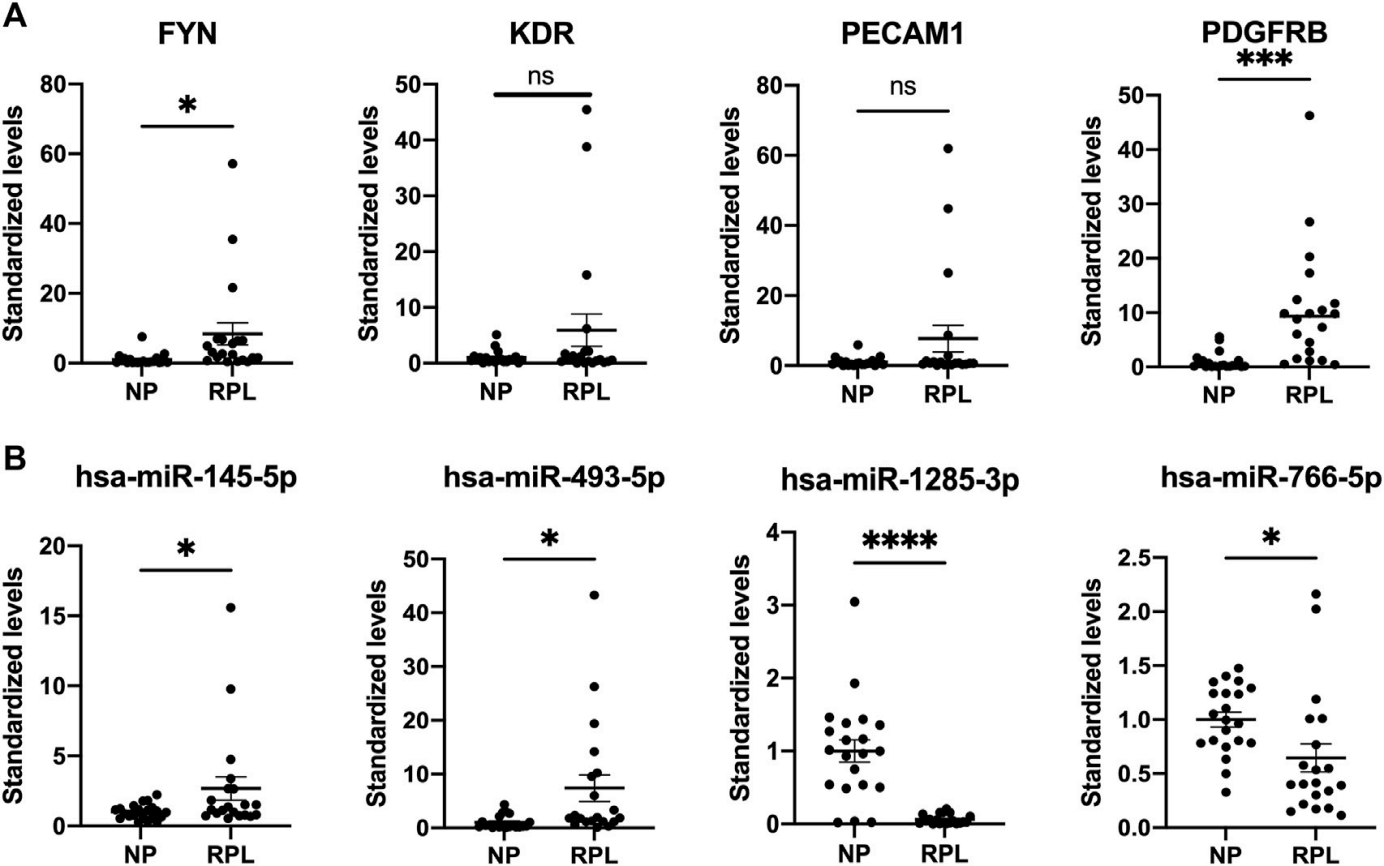
Validation of placental mRNAs and circulating miRNAs in the NP and RPL groups. mRNAs **(A)** and miRNAs **(B)** were confirmed by qPCR. The expression trends of candidate mRNAs and miRNAs were consistent with the sequencing results. Data are presented as 2−ΔΔct values (mean ± SEM). *p < 0.05, ***p < 0.001, and ****p < 0.0001; ns, not significant.

We selected 4 hub genes and 4 central differentially expressed miRNAs and increased the sample size to 20 RPL patients and 21 healthy controls to validate our RNA-seq data using qRT-PCR (Figure 8). The experimental results showed that the levels of FYN and PDGFRB mRNA were elevated in the placenta, which was consistent with the RNA-seq data. In addition, the levels of KDR and PECAM1 mRNA showed the same trends as the RNA-seq results but were not significant. MiR-145-5p and miR-493-5p were significantly upregulated in the plasma of RPL patients. In addition, miR-1285-3p and miR-766-5p were downregulated in RPL patients.

## Discussion

In this study, we characterized the circulating miRNA profile in the plasma and the mRNA profile in the placenta of patients with RPL. This study used a comprehensive approach to elucidate potential upstream regulators of key molecular pathways related to RPL by combining circulating miRNA and placental mRNA profiles. To our knowledge, this is the first study to integrate these two profiles from the same set of patients with RPL. In conclusion, our results show that the miRNA profile reflects the state of patients and is a potential noninvasive biomarker of RPL. Furthermore, alterations in plasma miRNA and placental mRNA expressions suggest the role of miRNAs in placental function in RPL.

In recent years, miRNAs have attracted extensive attention because of their high stability in circulating body fluids (including whole blood, plasma, serum, and saliva). Many ubiquitous and placenta-specific miRNAs are expressed and regulate trophoblast differentiation, proliferation, apoptosis, invasion/migration, and angiogenesis, suggesting that miRNAs play a significant role in placental development (Fu et al., 2013). These miRNAs can be produced by trophoblasts of the placenta and secreted into the maternal circulation. These miRNAs are potential diagnostic and prognostic biomarkers for pregnancy-related complications, as well as illness occurrence. We found 108 differentially expressed miRNAs in the plasma of RPL patients in our study, and there were 77 upregulated and 31 downregulated miRNAs. The tissues and cells from which circulating miRNAs are derived are often unknown. Therefore, we believe that the expressions of candidate diagnostic miRNAs in the circulation and placenta, as well as the evidence of important regulatory pathways involved in the pathophysiology of pregnancy loss, provide valuable indirect support for evaluating the diagnostic potential of these factors. Through combined placental RNA-seq analysis, we identified 7 central differentially expressed miRNAs that may play a broad regulatory role in the placenta. Then, potential biomarker miRNAs of RPL were further screened in larger samples (20 RPL patients and 21 NP controls) by real-time PCR. The results showed that in RPL patients, 2 miRNAs (miR-145-5p and miR-493-5p) were upregulated, and 2 miRNAs (miR-1285-3p and miR-766-5p) were downregulated. Our review of the literature revealed that these miRNAs, which had been replicated in more than one study, were expressed in the placenta (Lv et al., 2019; Inno et al., 2021; Smith et al., 2021). The four potential biomarker miRNAs identified in our study have never been linked to RPL before. The expression of miR-145-5p was increased in the placentas of patients with PE (Zhou et al., 2021). Mir-145-5p overexpression inhibited HTR-8/SVneo cell motility and invasion by negatively regulating Cyr61 expression through interactions with its 3′-untranslated region (UTR) (Wen et al., 2018). MiR-493-5p inhibits glioma progression by decreasing E2F3-mediated P53 and PI3K/AKT pathway dysfunction (Liu H. et al., 2022). Some studies have shown that mir-493-5p overexpression plays a negative regulatory role in esophageal cancer, osteosarcoma, cervical cancer, nonsmall cell lung cancer, and other malignancies by inhibiting cell proliferation/migration/invasion (Feng et al., 2021; Wang et al., 2021; Liu Q. et al., 2022; Huang et al., 2022). Downregulation of miR-1285-3p reversed the effects of weak expression of circRNA_400029 on the progression and apoptosis of cervical cancer cells (Ma et al., 2022). MiR-1285-3p overexpression promotes the development, metastasis, and invasion of a range of malignant tumors, including cervical cancer, lung adenocarcinoma, and colorectal cancer (Villanova et al., 2020; Zhang et al., 2021; Ma et al., 2022). Studies have shown that miR-766-5p can participate in the mitochondrial apoptotic pathway by targeting the 3′UTRs of BAX, BAK, and BOK, and the overexpression of miR-766-5p in SW480 cells has been shown to reduce cell death and improve viability (Dokanehiifard et al., 2020). Rapid proliferation, invasion, and migration of placental trophoblasts are required for effective embryo implantation and placental development (Brosens et al., 2022). These findings suggest that circulating miRNAs may be involved in RPL pathogenesis by targeting migration-, apoptosis-, and angiogenesis-related gene functions.

A total of 140 overlapping genes (overlapping between plasma miRNA target genes and actual placental disorder genes) were identified. This resulted in a total of 192 miRNA–mRNA interactions between 98 miRNAs and 140 target genes. In addition, using PPI analysis, we further identified the top 10 hub genes. In a consistent manner, the functional enrichment analysis of hub genes in placental villi showed that the regulated genes were mainly related to the regulation of cell proliferation, angiogenesis, cell migration, and focal adhesion, which are important processes involved in placental and fetal development. Studies have demonstrated that FYN expression, which is elevated at the fetomaternal interface of abortion-prone mice and RPL patients, plays a role in the regulation of fetomaternal immune tolerance by encouraging the expansion of Th17 cells and the expression of proinflammatory factors (Khan et al., 2014; Liu et al., 2016). A FYN inhibitor overcomes the aberrant inflammatory state and embryo resorption caused by LPS (Liu et al., 2016). KDR, also known as vascular endothelial growth factor receptor 2 (VEGFR2), was demonstrated to be elevated in the placenta of pathological pregnancy diseases (early pregnancy loss, PE) in many studies (Kumazaki et al., 2002; Plaisier et al., 2009; Fang et al., 2013; Sundrani et al., 2013). As a significant mediator of VEGF biological effects, KDR is important in uterine decidual and embryonic angiogenesis during early pregnancy (Douglas et al., 2009; Fang et al., 2013). In addition, a previous study has demonstrated that KDR participates in trophoblast migration, invasion, and proliferation by binding to decorin (Khan et al., 2011). PECAM-1 (platelet endothelial cell adhesion molecule-1), commonly known as CD31, is the most prevalent membrane glycoprotein that is expressed constitutively on the vascular endothelium (Chantraine et al., 2012; Caligiuri, 2020). In an interesting way, we found a trend toward higher levels of PECAM-1 expression in the placental villi of RPL patients. Consistent with our findings, another scientific study has shown an increased number of arterial, venous, and lymphatic vessels in placentas of RPL patients, and RPL is related to the decrease in venous and lymphatic invasion by extravillous trophoblasts rather than the total number of vessels (Windsperger et al., 2017). In an important way, our comprehensive approach showed that the downregulation of plasma miRNAs resulted in the upregulation of targeted mRNAs in placental tissue. Recent studies have identified correlations between circulating miRNAs and tissue gene expression, which lends support to our approach and findings. MiRNAs released from cells, such as in biological fluids like blood and breast milk, are thought to be present in vesicle-encapsulated, protein-bound forms, such as microvesicles, exosomes, and apoptotic bodies (Ouyang et al., 2014; Chang et al., 2017; Morales-Prieto et al., 2020). An emerging concept is that these miRNAs can silence neighboring or distant cells, allowing for hormonal-like intercellular communication associated with a variety of BP (Ouyang et al., 2014). Placental or other tissue-derived exosomal miRNAs are transferred to the maternal circulation and regulate neighboring or distant cells. Circulating exosomes released by placental syncytiotrophoblasts contain syncytin-1 and syncytin-2 on their surfaces, which can bind to MFSD2A and ASCT2 receptors on the surface of placental trophoblasts and contribute to exosome uptake (Vargas et al., 2014). *In vivo* and *in vitro* findings suggest that placental exosomal miR-15a-5p isolated from maternal plasma suppresses trophoblast proliferation, invasion, and apoptosis by downregulating CDK1 expression and impairing PI3K/AKT signaling, which is linked to the progression of PE (Wang et al., 2020a). Combined with our findings, key processes in the placenta may be affected by circulating dysregulated miRNAs in RPL.

In particular, our investigation discovered a number of previously unreported plasma biomarker miRNAs of RPL. This discovery may be due to the thorough control of the included population of pregnant women with normal fetal CNV, which is missing in other studies (Yang et al., 2018; Jairajpuri et al., 2021). From the genetic perspective, embryonic numerical and structural chromosomal abnormalities are known genetic causes of RPL, accounting for more than 50% of miscarriages (Wang et al., 2020b; Gu et al., 2022). Excluding the abortion caused by such genetic factors may more clearly identify the unknown pathogenesis of RPL. In addition, the presence of cellular contaminants such as platelets or erythrocytes and hemolysis both affect plasma miRNA detection results, and these factors may also be responsible for biases between conclusions (McDonald et al., 2011; Grasedieck et al., 2013). Therefore, we call for an effort to standardize the conclusions of different studies and accelerate their use in clinical practice by establishing a standardized process (monitoring and standardizing of sample collection, handling and storage, detection, and analysis).

Moreover, the limitations of this study must be noted. First, all RPL patients and healthy controls were Chinese. Our findings may not be applicable to patients of different ethnicities. Second, this study included a small number of samples from which miRNA characteristics were obtained. The most crucial evaluation requirements for circulating miRNA biomarkers are high sensitivity and specificity, but a single miRNA is less accurate because the levels in RPL patients and healthy controls may overlap, increasing the risk of false negative or positive diagnoses (Wang et al., 2018). Therefore, further research with more participants is needed to obtain a baseline of candidates and thus verify a panel of miRNAs as biomarkers. Third, placental villi tissue contains a variety of cells, mainly trophoblast cells but also immune cells such as monocytes, macrophages, T cells, and NK cells (Suryawanshi et al., 2018; Pique-Regi et al., 2019). This is our limitation because we chose mixed sample transcriptome sequencing, which did not allow us to deeply investigate gene expression analysis at the cellular level. In addition, understanding pathways linking miRNA binding, mRNA transcription, protein translation, and placental function may prove to be an ambitious task due to the complexity of the networks involved, which needs to be prioritized in future research to better understand the potential epigenetic mechanisms of RPL.

In conclusion, our study provides comprehensive data on circulating miRNAs and placental tissue mRNAs in RPL and shows that circulating miRNAs may become reliable biomarkers of RPL and may be involved in the pathogenesis of RPL. These results suggest a direction for the further study of RPL-related miRNAs and mRNAs and provide potential therapeutic targets for RPL.

## Data Availability

The datasets presented in this study can be found in online repositories. The names of the repository/repositories and accession number(s) can be found below: NCBI—PRJNA851566.
